# Trends of Different Surgical Approaches in Patients With Single-Level Lumbar Isthmic Spondylolisthesis: A National Registry Analysis

**DOI:** 10.7759/cureus.34194

**Published:** 2023-01-25

**Authors:** Mayur Sharma, Nikhil Jain, Dengzhi Wang, Beatrice Ugiliweneza, Maxwell Boakye

**Affiliations:** 1 Neurosurgery, University of Louisville School of Medicine, Louisville, USA; 2 Neurosurgery, University of Minnesota School of Medicine, Minneapolis, USA; 3 Orthopedic Surgery, University of Missouri School of Medicine, Columbia, USA; 4 Neurosurgery, University of Louisville, Louisville, USA; 5 Kentucky Spinal Cord Injury Research Center, University of Louisville, Louisville, USA; 6 Health Management and Systems Science, University of Louisville, Louisville, USA

**Keywords:** surgery, practice patterns, national database, isthmic spondylolisthesis, trends

## Abstract

Background: A variety of surgical approaches (anterior vs. posterior vs. anterior and posterior) are available for Isthmic Spondylolisthesis (IS). The aim of our study was to analyze the pattern and 30-day outcomes in patients undergoing different surgical approaches for single-level IS.

Materials and methods: National Surgical Quality Improvement Program (NSQIP) database was queried using the ICD-9/10 and CPT 4^th^ edition, from 2012 to 2020. We included patients 18-65 years of age who underwent spine fusions for IS. Outcomes were a length of stay (LOS), discharge disposition, 30-day complications, hospital readmission, and complication rates.

Results: Of 1036 patients who underwent spine fusions for IS, 838 patients (80.8%) underwent posterior only, 115 patients (11.1%) underwent anterior-only fusions and the rest (8%) underwent combined anterior and posterior procedures. 60% of patients in the posterior-only cohort had at least one comorbidity compared to 54% of patients in anterior only and 55% of patients in the combined cohort. No statistically significant differences in terms of LOS (3 days each) and discharge to home (96% vs. 93% vs. 94%) were noted among the anterior-only, posterior-only and combined cohorts, p> 0.05. In terms of 30-day complication rates, combined procedures had slightly higher rates (13%) compared to anterior (10%) or posterior-only (9%) procedures.

Conclusion: Posterior-only fusions were performed in 80% of patients with IS. No differences in terms of LOS, discharge disposition to home, 30-day complications, hospital readmission and reoperation rates were noted across the cohorts.

## Introduction

Lumbar Isthmic spondylolisthesis (IS) is a type II spondylolisthesis according to the Wiltse-Newman classification [1] and occurs secondary to defects in pars-interarticularis or isthmus.

Surgical management is usually indicated for refractory pain and neurological deficits resulting from high-grade slips and failure of conservative management (physical therapy, NSAIDs, lifestyle modifications, etc.) Various surgical techniques such as pars repair, decompression, and fusion have been described. Fusion techniques for spondylolisthesis include posterolateral instrumented fusion (PLF) with/without decompression, Interbody fusion (posterior lumbar interbody fusion [PLIF], transforaminal lumbar interbody fusion [TLIF], anterior lumbar interbody fusion [ALIF]) with decompression, reduction alone or with PLF, circumferential fusion, trans-sacral fibular dowel graft with PLF, transvertebral interbody cage fixation, intra-sacral rods, reverse-Bohlman technique and Spondylectomy for spondylosis [2-17].

Interbody fusion is the most commonly performed approach in the US using a Nationwide Inpatient sample study (NIS) [18,19]. A recent systematic review (six studies, 397 patients) reported no significant difference between anterior and posterior approaches for IS in terms of fusion rates and clinical outcomes, however with higher complications using the anterior approach [17]. Similarly, another systematic review (six RCTs and nine observational studies) reported no significant difference between interbody fusion and posterior-only fusion for low-grade IS in terms of clinical outcomes and complication rates at three-year follow-ups [20]. Also, there was insufficient evidence regarding the efficacy of different interbody fusion techniques (ALIF, TLIF, and PLIF) and circumferential fusion for IS in this study [20]. Moreover, there is a paucity of literature focusing on patterns of surgical procedures and their impact on short-term clinical outcomes (30-day complications and readmission rates) using different surgical techniques in patients with IS using a large dataset depicting real-world practice.

In this study, we aim to analyze the pattern and impact of different surgical approaches (anterior vs. posterior vs. anterior and posterior fusion techniques for single-level fusions) on 30-day outcomes in patients with IS using the American College of Surgeons National Surgical Quality Improvement Program database ACS-NSQIP database. We analyzed the length of hospital stay (LOS), complications, discharge disposition, and readmissions among the cohorts at 30 days post-discharge. We hypothesize that anterior + posterior and anterior-only approaches for IS may result in a higher incidence of complications and delayed discharge dispositions in short term compared to posterior-only approaches for IS.

## Materials and methods

Data source

National Surgical Quality Improvement Program (NSQIP) is a nationally validated outcome-based database of over 700 hospitals introduced by the American College of Surgeons (ACS) to improve the quality of surgical care. This data is available for participating institutions for free. We used records from 2012 to 2020. This study is approved by the Institutional review board (IRB) at our institute.

Study cohort 

We included adult patients (18-65 years of age) with the diagnosis of Lumbar Isthmic spondylolisthesis using the International Classification of Disease, 9th Revision (ICD-9: 756.11 and 756.12) and 10th Revision (ICD-10: Q76.2) diagnosis codes. We decided to include this age range to exclude the effects of degenerative spine spondylolisthesis on IS and include patients with IS only. All the patients had to have undergone concurrent lumbar fusion anterior technique (Current Procedural Terminology, 4th edition [CPT-4 codes] 22558, 22586 excluding percutaneous posterior procedures CPT-4 22840, posterior technique [CPT-4 codes 22612, 22630, 22633]), or 360 degrees fusion both anterior and posterior including CPT 22840 for posterior percutaneous screws). To include patients who underwent only single-level procedure, we excluded codes for additional anterior (CPT-4: 22534, 22585) and posterior procedures (CPT-4: 22614, 22634) (Table 1).

**Table 1:** ICD-9/ICD-10/CPT codes used for extracting the data for patients with Lumbar Isthmic Spondylolisthesis (IS) who underwent anterior, posterior, or combined spine fusions.

	ICD-9 Code	ICD-10 Code	CPT-4th Code
Diagnosis codes			
Lumbar Isthmic spondylolisthesis	756.11, 756.12	Q76.2	
Procedure codes			
Anterior lumbar fusion			22558, 22586, excluding 22840 for posterior percutaneous screws
Posterior lumbar fusion			22612, 22630, 22633
Combined anterior + posterior fusion			(22558, 22586) +(22612, 22630, 22633) + 22840 for posterior percutaneous screws
Excluded codes			
Additional anterior and posterior procedure			22534, 22585; 22614, 22634

Patient characteristics

Demographics (age, sex, race, ethnicity) are recorded at the time of surgery. BMI was calculated from weight and height which are also noted at the surgery hospital admission. NSQIP documents the multiple comorbidities, for this study, comorbidity was dichotomized using two categories, no comorbidities or at least one comorbidity. 

Outcomes

Outcomes of interest were the length of hospital stay (LOS) following the index surgery, discharge disposition (home or not e.g. rehabilitation or skilled nursing facility) as well as 30-day complications, hospital readmission, and reoperation rates. Complications such as superficial or deep surgical site infection (SSI), Respiratory (pneumonia, pulmonary embolism, need for intubation, the requirement of the ventilator), Renal [insufficiency, acute renal failure (ARF), urinary tract infection (UTI)], Neurological (stroke or cerebrovascular accident with neurological deficit, coma, peripheral nerve injury), Cardio-vascular [cardiac arrest, Myocardial infarction (MI), bleeding transfusions], graft/prosthesis/flap failure, DVT/thrombophlebitis, Sepsis/septic shock. We dichotomized patients with at least one complication vs. no complications. 

Statistical analysis

Categorical patient characteristics and outcomes were summarized with frequency count with associated percentages and compared across groups using the Chi-Square test. Continuous variables were tested for normality using the Kolmogorov-Smirnov test. Those that were normally distributed were summarized with mean with standard deviation and compared across fusion technique groups using Analysis of Variance (ANOVA). Those not normally distributed were summarized with median with 1st and third quartiles and were compared across groups with the Brown-Mood test. The significance level was set to 0.05 and all tests were two-sided. Analyses were performed in SAS 9.4 (SAS Inc, Cary, NC).

## Results

Patient demographics and characteristics

**Figure 1:**
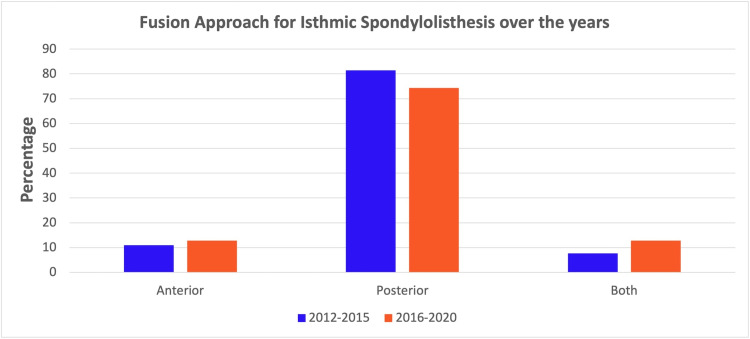
Bar graphs showing the trends of anterior only, posterior only and combined procedures for single level isthmic spondylolisthesis from 2012 to 2020.

A cohort of 1036 patients 18-65 years of age with the diagnosis of IS who underwent spine fusions were identified from the database from 2012-2020. Of these, the majority (80.8%, n=838) underwent posterior-only fusions, whereas 11.1% (n=115) underwent anterior-only fusions and 8.0% (n=83) underwent combined anterior and posterior procedures for IS. The use of anterior-only and combined procedures gradually increased from 9% and 4% in 2012 to 11% and 22% in 2020. However, use of posterior-only procedures gradually declined from 87% (2012) to 67% (2020) (Figure 1).

Overall, the median age of the patients was 52 years, and more than half of the patients were females (56%) and Caucasians (82%). Hispanics constituted only 6% of all the patients. Median BMI was 24 across the cohorts and 59% of the patients had at least one comorbidity.

Patients in the anterior-only cohort were slightly younger (median age 50 years) compared to the posterior only or combined cohort (median age 52 years each), however this difference was not significant. Females represented 63% of the combined cohort compared to 49% of anterior only and 56% of posterior only cohort. Patients who underwent combined procedures had slightly better BMI compared to other cohorts (23 vs. 24 each). 60% of patients in the posterior only cohort had at least one comorbidity compared to 54% of patients in anterior only and 55% of patients in combined cohort. No statistically significant difference was noted in terms of any of the demographic parameters across the cohorts (Table 2). Since all the characteristics were statistically comparable, covariance adjustment methods such as regression, propensity or exact matching were not used in the analysis.

**Table 2:** Comparison of demographics of patients with lumbar isthmic spondylolisthesis (IS) who underwent anterior, posterior, or combined spine fusions, 2012-2017.

Variables		All	Anterior	Posterior	Both	P-value
		(n = 1036)	(n = 115)	(n = 838)	(n = 83)	
Age, median (Q1-Q3)		52 (43 - 59)	50 (42 - 55)	52 (44 - 59)	52 (42 - 59)	0.2009
Sex, Female, n (%)		576 (56%)	56 (49%)	468 (56%)	52 (63%)	0.1414
Race	White, n (%)	854 (82%)	97 (84%)	685 (82%)	72 (87%)	
	Black, n (%)	77 (7%)	10 (9%)	66 (8%)	1 (1%)	0.1596
	Other, n (%)	105 (10%)	8 (7%)	87 (10%)	10 (12%)	
BMI, median (Q1-Q3)		24 (21 - 28)	24 (21 - 28)	24 (21 - 28)	23 (19 - 27)	0.4761
Comorbidity, at least one, n (%)		613 (59%)	62 (54%)	505 (60%)	46 (55%)	0.3308

Outcomes at index hospitalization and 30 days after the procedure

No statistically significant differences in terms of LOS (three days each) and discharge to home (96% vs. 93% vs. 94%) were noted among the anterior only, posterior only and combined cohorts, respectively, p > 0.05. In terms of 30-day complication rates, combined procedures had slightly higher rates (13%) compared to anterior (10%) or posterior-only (9% each) procedures, with no statistical difference. Posterior-only procedures had lower 30-day hospital readmission and reoperations rates (4% and 3%) compared to anterior only (10% each) or combined procedures (13% each) (Table 3).

**Table 3:** Hospital and 30-day outcomes for patients who underwent fusion for lumbar isthmic spondylolisthesis

Outcomes	All	Anterior	Posterior	Both	P-value 3 groups
	(n = 1036)	(n = 115)	(n = 838)	(n = 83)	
Hospital LOS, Median (Q1-Q3)	3 (2 - 4)	3 (2 - 4)	3 (2 - 4)	3 (2 - 4)	0.9074
Discharge Home, n (%)	971 (94%)	110 (96%)	783 (93%)	78 (94%)	0.6526
30-day complications, n (%)	93 (9%)	11 (10%)	75 (9%)	<11 (<13%)	0.9611
30-day hospital readmission, n (%)	36 (3%)	<11 (<10%)	31 (4%)	<11 (<13%)	0.4963
30-day reoperation, n (%)	32 (3%)	<11 (<10%)	27 (3%)	<11 (<13%)	0.5792

## Discussion

In our study, we found that anterior-only and combined procedures were increasingly being performed for IS; however, the posterior-only procedure was performed in most patients. Also, a posterior-only procedure was performed in slightly older patients with comorbidities. There were no differences in terms of LOS, discharge disposition, and 30-day outcomes (complications, re-operation, and readmissions) among the groups in this study. 

In our study, we found that the median age of the patients who underwent anterior-only procedures was 50 years compared to 52 years for patients who underwent either posterior-only or combined procedures with no statistical difference among the groups. Similarly, Alhammoud et al. [17], in a systematic review and meta-analysis of six studies, reported median age of 44 years for anterior-only approaches, 49 years for posterior-only approaches, and 48 years for combined approaches. However, anterior-only approaches were reported in only one study in this meta-analysis. Another retrospective study reported a mean age of 51 years for ALIF-only procedures and 47 years for TLIF-only procedures in patients with single-level L5-S1 IS with no differences across the cohorts [21]. In contrast, a Nationwide inpatient sample (NIS) database study reported mean age of 49 years for anterior-only approaches, compared to 56 years for posterior-only and 47 for combined procedures with statistically significant differences across the cohorts (1998-1999) [18]. This study also reported a slight increase in the age of the patients who underwent procedures during the years 2010-2011 (54 years for anterior only, 63 years for posterior only, and 57 years for combined procedures). This difference may be related to the type of database and the number of patients in each cohort, which may be statistically different with no significant clinical implications. 

Clinical guidelines developed by the North American Spine Society (NASS) in 2016 for managing adult patients with IS provided grade A recommendations for either posterolateral or 360-degree fusion in patients with low-grade IS [22]. They also recommended that 360-degree fusion provide higher radiographic fusion rates compared with posterolateral fusion (Grade A recommendation). However, the guidelines committee reported that there was insufficient or conflicting evidence for any differences in clinical outcomes following either 360-degree fusion or posterolateral fusion for low-grade IS [22]. In terms of stand-alone ALIF procedures for low-grade IS, this committee recommended that such procedures may be considered based on the clinical setting (Grade C recommendation). Moreover, since the introduction of the negative impact of positive sagittal balance on health status outcomes in 2005 [23], a number of studies have shown that the maintenance of post-operative global sagittal balance is critical in optimizing outcomes [24,25]. A retrospective study (n=66) comparing combined and TLIF procedures for IS showed that combined procedures resulted in significantly greater improvement in segmental lordosis and disc height with better functional outcome scores compared to TLIF [26]. These results were reiterated by another recently published study which showed that combined procedures resulted in greater segmental lordosis compared to TLIF at both the first visit (mean: 26 days, 11.3° vs 1.3°) and at the last visit (mean: 410 days, 9.6° vs 0.2°) [21]. In terms of disc height, this study also reported a greater increase in disc height with combined procedure compared to TLIF at both first (9.6 vs 5.5mm) and final follow-up (8.7 vs 3.6mm), which also resulted in improved patient-reported outcome measures [21].

In our study, we found that almost four-fifth of the patients underwent posterior-only fusions for IS during the study period. However, the use of anterior-only and combined procedures gradually increased from 9% and 4% in 2012 to 11% and 22% in 2020, with a corresponding decrease in the posterior-only procedures (87% to 67%) which may be attributed to the increasing focus on achieving good sagittal alignment and radiographic fusion [27]. Interestingly, in our study, 11% of patients underwent anterior-only procedures for IS, despite the fact that stand-alone ALIF for IS with high pelvic incidence may result in instrumentation failure, pseudoarthrosis, and sacral fractures [28, 29]. Another study using the NIS database 1998-2011, reported that the annual rates of anterior-only fusions increased 2.65 times, posterior-only fusions increased 4.33 times, and combined procedures increased 2.93 times during the study period [18]. This discrepancy in the rates of posterior-only procedures may be attributed to different databases and study periods used in the two studies.

In terms of 30-day complication rates, we found that combined procedures had slightly higher rates (13%) compared to anterior (10%) or posterior-only (9%) procedures. Our results are similar to the study published using the NIS database, which reported complication rates of 18.86% for combined procedures compared to 2.56% for posterior-only procedures [18]. This difference in complication rates for posterior-only procedures may be attributed to the differences in the databases and the way the data was recorded. Similarly, another systematic review reported a higher number of complications with anterior procedures compared to posterior procedures (39 vs. 30) [17]. In our study, the median LOS was 3 days, which was similar across all the surgical techniques, which was similar to the mean LOS of 3.18 (anterior only), 3.47 (posterior only), and 4.31 (combined procedures) using the NIS database [18]. Also, 30-day reoperation rates in our study were 3% in the posterior-only cohort compared to 10% in the anterior-only and 13% in the combined cohorts. A recent systematic review reported almost double the re-operation rates for posterior-only approaches compared to the anterior approaches (8% vs. 4% at 27.2 and 29.4 months of follow-up respectively) [17]. This discrepancy may be related to only the 30-day assessment in our study.

Future implications of the research

The results of our study showed the current practice patterns in terms of surgical approaches in patients with IS using real-world databases. There were no differences in 30-day complications, LOS, discharge disposition, and readmissions based on surgical approaches in patients with IS. These findings are likely to help spine surgeons in selecting an appropriate surgical strategy that they are comfortable with and counsel patients pre-operatively. Future prospective studies are needed to confirm these findings using high-level evidence.

Strength and limitations

NSQIP database provides clinical data for an individual diagnosis and intervention across the participating sites in the US. This database is useful to study the patterns of treatment paradigms and compare 30-day outcomes for rare pathologies across the US in a large cohort of patients. 

Limitations of using such a database include the retrospective observational design of the study, selection bias, and confounding bias from unknown factors. ICD and CPT codes were utilized to extract the data, therefore coding errors, missing data, and variability in coding data should be considered. Clinical data such as patient symptoms and imaging findings (slip angle, degree of kyphosis, etc.) that resulted in choosing a particular surgical strategy cannot be extracted using this database. Also, procedures such as ALIF and LLIF cannot be separated, as they share the same CPT code, however, since L5-S1 is the commonly involved level with IS and ALIF is the preferred approach for that level instead of LLIF; therefore, this limitation is unlikely to affect our results. Also, we cannot tease out different posterior-only subgroups such as PLIF or TLIF for IS using this database. Patient satisfaction, individual patient function, and long-term outcomes cannot be analyzed using this database. Also, we only looked at short-term (30-day) outcomes and therefore outcomes such as fusion rates, re-operation rates, and long-term complications were not analyzed in this study. Also, given the small number of patients with outcomes, the effect of different covariables on outcomes was not analyzed. Therefore, the findings of this study need to be interpreted considering these limitations. Nevertheless, our study provides insight from the real-world clinical data showing the patterns and impact of different surgical strategies in a large cohort of patients with IS.

## Conclusions

Anterior-only and combined procedures are increasingly being performed for IS, with decreasing use of posterior-only procedures during the study period. Posterior-only fusions were performed in 80% of patients with IS in this study. No differences in terms of LOS, discharge disposition to home, 30-day complications, hospital readmission, and reoperation rates were noted across all three cohorts. Based on this study, a surgeon can choose whichever surgical approach s/he determines is best, with no impact on the index and 30-day outcome measures. These findings may assist in clinical decision-making and preoperative counseling of patients.

## References

[1] Wiltse LL, Newman PH, Macnab I (1976). Classification of spondylolisis and spondylolisthesis. Clin Orthop Relat Res.

[2] Meyerding HW (1956). Spondylolisthesis; surgical fusion of lumbosacral portion of spinal column and interarticular facets; use of autogenous bone grafts for relief of disabling backache. J Int College Surg.

[3] Stoker GE, Buchowski JM, Zebala LP, Lenke LG, Bridwell KH (2011). High-grade spondylolisthesis: surgical management in the pediatric and young adult patient. ArgoSpine News J.

[4] Kasliwal MK, Smith JS, Kanter A, Chen CJ, Mummaneni PV, Hart RA, Shaffrey CI (2013). Management of high-grade spondylolisthesis. Neurosurg Clin N Am.

[5] Macagno AE, Hasan S, Jalai CM (2016). "Reverse Bohlman" technique for the treatment of high grade spondylolisthesis in an adult population. J Orthop.

[6] Hire JM, Jacobs JM, Bundy JV, DeVine JG (2015). A modified Bohlman technique using a novel implant for treatment of high-grade spondylolisthesis. J Neurosurg Spine.

[7] Rindler RS, Miller BA, Eshraghi SR, Pradilla G, Refai D, Rodts G, Ahmad FU (2016). Efficacy of transsacral instrumentation for high-grade spondylolisthesis at L5-S1: a systematic review of the literature. World Neurosurg.

[8] Bozkus H, Dickman CA (2004). Transvertebral interbody cage and pedicle screw fixation for high-grade spondylolisthesis. Case report. J Neurosurg.

[9] Abdu WA, Wilber RG, Emery SE (1994). Pedicular transvertebral screw fixation of the lumbosacral spine in spondylolisthesis. A new technique for stabilization. Spine (Phila Pa 1976).

[10] Lakshmanan P, Ahuja S, Lewis M, Howes J, Davies PR (2009). Transsacral screw fixation for high-grade spondylolisthesis. Spine J.

[11] Bouyer B, Bachy M, Courvoisier A, Dromzee E, Mary P, Vialle R (2014). High-grade lumbosacral spondylolisthesis reduction and fusion in children using transsacral rod fixation. Childs Nerv Syst.

[12] Bollini G, Jouve JL, Launay F, Glard Y, Jacopin S, Blondel B (2011). High-grade child spondylolisthesis: a custom-made canulated screw to treat the so-called double instability. Orthop Traumatol Surg Res.

[13] Sasso RC, Shively KD, Reilly TM (2008). Transvertebral Transsacral strut grafting for high-grade isthmic spondylolisthesis L5-S1 with fibular allograft. J Spinal Disord Tech.

[14] Laurent LE, Osterman K (1976). Operative treatment of spondylolisthesis in young patients. Clin Orthop Relat Res.

[15] Smith MD, Bohlman HH (1990). Spondylolisthesis treated by a single-stage operation combining decompression with in situ posterolateral and anterior fusion. An analysis of eleven patients who had long-term follow-up. J Bone Joint Surg Am volume.

[16] Sharma M, Aljuboori Z, Clouse JW, Rodgers R, Altstadt T (2019). Sacro-iliac joint fusion system for high-grade Spondylolisthesis using "Reverse Bohlman technique": a technical report and overview of the literature [PREPRINT]. World Neurosurg.

[17] Alhammoud A, Schroeder G, Aldahamsheh O, Alkhalili K, Lendner M, Moghamis IS, Vaccaro AR (2019). Functional and radiological outcomes of combined anterior-posterior approach versus posterior alone in management of isthmic spondylolisthesis. A systematic review and meta-analysis. Int J Spine Surg.

[18] Thirukumaran CP, Raudenbush B, Li Y, Molinari R, Rubery P, Mesfin A (2016). National trends in the surgical management of adult lumbar isthmic spondylolisthesis: 1998 to 2011. Spine (Phila Pa 1976).

[19] Alomari S, Judy B, Sacino AN (2022). Isthmic spondylolisthesis in adults… A review of the current literature. J Clin Neurosci.

[20] Noorian S, Sorensen K, Cho W (2018). A systematic review of clinical outcomes in surgical treatment of adult isthmic spondylolisthesis. Spine J.

[21] Lightsey HM 4th, Pisano AJ, Striano BM (2022). Alif versus TLIF for L5-S1 isthmic spondylolisthesis: Alif demonstrates superior segmental and regional radiographic outcomes and clinical improvements across more patient-reported outcome measures domains. Spine (Phila Pa 1976).

[22] Kreiner DS, Baisden J, Mazanec DJ (2016). Guideline summary review: an evidence-based clinical guideline for the diagnosis and treatment of adult isthmic spondylolisthesis. Spine J.

[23] Glassman SD, Bridwell K, Dimar JR, Horton W, Berven S, Schwab F (2005). The impact of positive sagittal balance in adult spinal deformity. Spine (Phila Pa 1976).

[24] Ochtman AE, Kruyt MC, Jacobs WC, Kersten RF, le Huec JC, Öner FC, van Gaalen SM (2020). Surgical restoration of sagittal alignment of the spine: correlation with improved patient-reported outcomes: a systematic review and meta-analysis. JBJS Rev.

[25] Ogura Y, Shinozaki Y, Kobayashi Y (2019). Impact of sagittal spinopelvic alignment on clinical outcomes and health-related quality of life after decompression surgery without fusion for lumbar spinal stenosis. J Neurosurg Spine.

[26] Tye EY, Tanenbaum JE, Alonso AS, Xiao R, Steinmetz MP, Mroz TE, Savage JW (2018). Circumferential fusion: a comparative analysis between anterior lumbar interbody fusion with posterior pedicle screw fixation and transforaminal lumbar interbody fusion for L5-S1 isthmic spondylolisthesis. Spine J.

[27] Wang SJ, Han YC, Liu XM, Ma B, Zhao WD, Wu DS, Tan J (2014). Fusion techniques for adult isthmic spondylolisthesis: a systematic review. Arch Orthop Trauma Surg.

[28] Jaeger A, Giber D, Bastard C, Thiebaut B, Roubineau F, Flouzat Lachaniette CH, Dubory A (2019). Risk factors of instrumentation failure and pseudarthrosis after stand-alone L5-S1 anterior lumbar interbody fusion: a retrospective cohort study. J Neurosurg Spine.

[29] Phan K, Mobbs RJ (2015). Sacrum fracture following L5-S1 stand-alone interbody fusion for isthmic spondylolisthesis. J Clin Neurosci.

